# A Visit to the Hospitals of Montreal—II

**Published:** 1894-10-27

**Authors:** 


### The Institutional Workshop.

### CANADIAN HOSPITALS.

By Our Own Correspondent,

### A VISIT TO THE HOSPITALS OF MONTREAL.—II.

The Hôtel Dieu of to-day differs from that of two centuries ago. The once formidable Iroquois are represented by a handful of lazy men to whom the title of "braves" would seem misapplied, dressed in ordinary farmer costume, and depressed looking women who sell specimens of gaudy and inartistic beadwork to the newly-arrived tourist. They live safely outside the city, on the other side of the St. Lawrence, and care more for the privilege of travelling for half-fare on the Canadian Pacific Railway, than for the scalps of many pale-faces. Their reservation, Cachenaga, situated on the bank of the river, might be a fertile spot, if they did not retain so much of Indian dignity as to make them despise the arts of husbandry.

But though the sisters have no longer arrow wounds to treat, though no longer does an ungrateful Indian, healed of his hurts, attempt to strangle his nurse, the work of the Hôtel Dieu has increased with every decade. The French still form three-fourths of the population of the Province of Quebec, and though the sisters make no distinction of race or creed in admitting the sick, it is natural that the sick themselves should often choose to go where creed and language harmonise with their own. Of English-speaking patients the majority are Catholics of Irish descent, although a number of Protestants also are to be found in the white-curtained beds, which instead of the conventional number of the ordinary hospital, are distinguished from each other by the names of the many saints of the Church. The hospital no longer stands on the original site. The log building, burnt down and rebuilt more than once, gave place to a permanent erection of stone, and though wharves and warehouses surrounded them, the sisters were content. But as sanitary science became more widely recognised the doctors became dissatisfied with the cramped and ill-ventilated site, and in 1861 persuaded the nuns to remove to a new hospital built on a piece of ground just outside the town and near the mountain, which had been given to them in 1730 by Benoit and Gabriel Basset. In 1861 the remains of Mademoiselle Mance and 176 nuns were transferred to the chapel of the new institution.

The present Hôtel Dieu centres in this chapel—in truth a large and handsome church dedicated to St. Joseph—from which branches on the left the convent, and on the right the hospital. The institution contains 230 beds in all. There are, however, only four wards, two male and two female, and a considerable number of private rooms for paying patients. The paying patient is a recognised institution in all American hospitals, and much surprise is expressed at the small provision that is made for him in Britain. In some cases, it seemed to me, the American tendency may be to limit unduly the charitable work of the hospital, but at the Hôtel Dieu this certainly was not so. But the paying patient being thus an accepted feature of hospital life, he is specially looked after. The private rooms are comfortably and tastefully furnished. Besides the bed there is a bureau, washstand, and table of walnut or maple, a well-upholstered couch, a rocking-chair, bright hued draperies, and often embroidered scarfs, needless, but dainty, for the dressing-table. In short, these rooms are bright, pleasant, and home-like; there is hospital cleanliness and hospital care, but the suggestion of the "institution" is banished.

By chance I visited the Hôtel Dieu on Corpus Christi day. The general Corpus Christi procession walked through the streets of Montreal on the Sunday following, but as these cloistered sisters could not join in it they had a procession of their own through the wards and the convent. An altar had been raised in the largest male ward, a long room holding 44 beds. As I gazed down the long vista of white-curtained beds, of the old-fashioned tent shape, all shut in with spotless lawn, and thought of the helpless creatures whom they held—though all that could stir were up and dressed today—I grew nervous at the sight of the muslin drapings and twinkling candles on the altar. A single spark, one feared, might set so much ablaze. But no accident occurred. The procession began with a long string of children, first boys bearing a banner, then a more numerous array of girls. The youngest of these were dressed in scarlet frocks, the elder ones in black gowns, and all wore white veils over their heads. A number of women, similarly dressed, followed the children. Then came the Host, borne under its silk and gold-fringed canopy by a priest in magnificent vestments, supported by two others hardly less richly dressed. With these came choristers and acolytes, attired in white and red. Last followed the nuns themselves, the novices in white veils, the sisters with the black veils, which are generally, for convenience, thrown back from the face, now pulled over their brows. All sank to their knees, while a short service was intoned, and when the procession moved on again it was joined by a number of convalescents, to some of whom the walk through ward and corridor seemed enough of an effort. Again at stated spots all knelt in prayer, and finally the procession filed into the church for a last service there.

As I followed with the rest I noticed the mottoes at every corner, "Jesus our Shepherd," "Jesus our Friend"—every attribute of the Christ was there in turn, up to the culmination, "Jesus our All." Above one doorway was a beautiful plaster group representing the child Christ walking between his parents, and beneath the figures the inscription (in French, as everything is about the Hôtel Dieu), "Oh, blessed Trinity of earth, lead us to the Trinity above!" A strange and touching aspiration, I could not but think, for women who sought the path to heaven away from the heaven-created road of home, though their happy, energetic looking countenances gave no suggestion of discontent with the way they had chosen to traverse. But the inscription which, I think, charmed me most of all was this, printed on the wall of the well-arranged and beautifully-lighted operating theatre, which can hold 256 students: "If the surgeon is to attend to everything, never go astray, forget nothing, he ought to know how to respect the natural indications given by the Great Master." Is there no other theatre in the world where such a warning would be fitly placed?

